# Neoadjuvant EGFR-TKI Therapy for EGFR-Mutant NSCLC: A Systematic Review and Pooled Analysis of Five Prospective Clinical Trials

**DOI:** 10.3389/fonc.2020.586596

**Published:** 2021-01-12

**Authors:** Li Sun, Yi-Jia Guo, Jun Song, Yan-Ru Wang, Shu-Ling Zhang, Le-Tian Huang, Jian-Zhu Zhao, Wei Jing, Cheng-Bo Han, Jie-Tao Ma

**Affiliations:** Department of Oncology, Shengjing Hospital of China Medical University, Shenyang, China

**Keywords:** neoadjuvant, non-small cell lung cancer, efficacy, safety, epidermal growth factor receptor-tyrosine kinase inhibitors

## Abstract

**Purpose:**

The role of neoadjuvant epidermal growth factor receptor (EGFR)-tyrosine kinase inhibitor (TKI) targeted therapy for patients with EGFR-mutant non-small cell lung cancer (NSCLC) has not been clarified. A pooled analysis of prospective clinical trials was conducted to evaluate the efficacy and safety of neoadjuvant EGFR-TKI therapy.

**Methods:**

The PubMed, Embase, Web of Science, and Cochrane Library databases, as well as meeting abstracts were searched for prospective clinical trials evaluating the efficacy and safety of neoadjuvant EGFR-TKI for treatment of EGFR-mutant NSCLC. The main outcomes included the objective response rate (ORR), downstaging rate, surgical resection rate (SRR), pathologic complete response (pCR) rate, progression-free survival (PFS), and adverse events.

**Results:**

A total of five, phase II, prospective, clinical trials involving 124 patients with resectable or potentially resectable EGFR-mutant NSCLC treated with neoadjuvant erlotinib or gefitinib treatment were included in this pooled analysis. The median neoadjuvant medication time was 42 (range, 21–56) days and the median time of response evaluation was 45 (range, 42–56) days. The pooled ORR was 58.5% [95% confidence interval (CI), 45.5%–71.8%] and the surgical resection and complete resection (R0) rates were 79.9% (95% CI, 65.3%–94.5%) and 64.3% (95% CI, 43.8%–84.8%), respectively. In the stage IIIA subgroup (n = 68), the pooled ORR, SRR, and R0 rate were 51.4%, 72.9%, and 57.0%, respectively, while the downstaging and pCR rates were 14.0% and 0.0%, respectively. The pooled median PFS and overall survival were 13.2 and 41.9 months, respectively. Of the most common grade 3/4 adverse events in the overall group, the incidences of hepatotoxicity and skin rash were 5.3% and 14.7%, respectively. The most commonly reported postoperative complications were lung infection, arrhythmia, and pneumothorax.

**Conclusion:**

Neoadjuvant EGFR-TKI therapy provides a feasible treatment modality for patients with resectable or potentially resectable EGFR-mutant NSCLC, with satisfactory surgical outcomes and low toxicity. Although further phase III clinical trials are needed to confirm these findings, it is necessary to explore the feasibility of a more effective EGFR-TKI combination neoadjuvant therapy given the modest downgrade and pCR rates for EGFR-TKI alone.

## Introduction

Lung cancer is the most common malignancy and the leading cause of cancer-related deaths worldwide. Non-small cell lung cancer (NSCLC) accounts for 80%–85% of all lung cancers (1). For patients with early resectable NSCLC, surgery remains the cornerstone of treatment. Although resection can achieve good local control, the rates of regional recurrence and distant metastasis remain very high. As preoperative systemic therapy has the potential to reduce disease stage and facilitate surgical resection, in addition to the value of drug sensitivity tests to guide postoperative treatment, a series of studies of neoadjuvant systematic therapies, including chemotherapy, targeted therapy, and immunotherapy, have been conducted to explore the possibility of improving the cure rate and survival rate (2–5). Multiple meta-analyses based on large-scale prospective randomized controlled trials (RCTs) confirmed a modest survival benefit of preoperative chemotherapy for NSCLC (6, 7).

For patients with oncogenic driver (e.g., epidermal growth factor receptor [EGFR], anaplastic lymphoma kinase, and proto-oncogene ROS1)-positive advanced NSCLC, targeted therapy with small molecule tyrosine kinase inhibitors (TKIs) has greatly improved the therapeutic outcomes and has become the first-line treatment standard. As compared to chemotherapy, EGFR-TKIs significantly improve the objective response rate (ORR) and progression-free survival (PFS) for patients with EGFR-mutant advanced NSCLC (8–11). Beyond that, for EGFR-mutant stage II or III NSCLC patients, as compared with chemotherapy/placebo, postoperative adjuvant EGFR-TKI therapy significantly prolongs disease-free survival (DFS), with a 3-year DFS rate of 34%–80% in the EGFR-TKI group versus 20%–28% in the chemotherapy/placebo group (12–14).

In view of the robust anti-tumor activity and tumor remission rate of EGFR-TKI against EGFR-mutant advanced diseases, many recent studies have explored the feasibility of neoadjuvant EGFR-TKI therapy for the treatment of NSCLC. However, most of these studies were single arm prospective clinical trials. A prospective phase II RCT launched by the Chinese Thoracic Oncology Group (CTONG) 1103 reported at the 2018 European Society for Medical Oncology (ESMO) meeting that, as compared with neoadjuvant chemotherapy, neoadjuvant EGFR-TKI therapy for patients with EGFR-mutant stage IIIA NSCLC had a significant advantage in PFS (21.5 vs. 11.4 months; hazard ratio = 0.39; *p* < 0.001) (5). Therefore, the aim of this pooled analysis based on prospective clinical trials was to evaluate the efficacy and safety of neoadjuvant EGFR-TKI therapy in patients with resectable or potentially resectable EGFR-mutant NSCLC, and to provide a basis for decision-making on neoadjuvant EGFR-TKI therapy.

## Materials and Methods

### Search Strategy

This pooled analysis was conducted in accordance with the guidelines of the Preferred Reporting Items for Systematic Review and Meta-analyses (PRISMA) (15). The PubMed, Embase, Web of Science, and Cochrane Library databases, as well as meeting abstracts from the American Society of Clinical Oncology, ESMO, European Lung Cancer Conference, and World Conference on Lung Cancer were searched for relevant trials using the following search terms: “lung cancer” AND “EGFR” AND “neoadjuvant” OR “induction” OR “preoperative”. The reference lists of the enrolled studies were carefully scanned to ensure that all relevant literature was retrieved. The final literature search was performed on March 20, 2020.

### Inclusion Criteria

The inclusion criteria were as follows: 1) prospective studies that evaluated the efficacy or safety of preoperative EGFR-TKI for resectable or potentially resectable NSCLC with an EGFR-sensitive mutation; 2) outcomes that included at least one of these endpoints: ORR, PFS, DFS, overall survival (OS), surgery resection rate (SRR), complete (R0) resection rate, downstaging rate, pathologic complete response (pCR) rate, or adverse events (AEs); and 3) the inclusion of ≥ 10 cases.

### Data Extraction

Two authors screened the authorship and titles to extract preliminary eligible studies and exclude duplicate studies. Then, the titles, abstracts, and full text of the retrieved articles were further screened to identify studies that met the inclusion criteria. Two authors independently extracted data from all eligible studies, which included 1) the name of the first author and the publication year; 2) study characteristics, including patient characteristics, disease stage, EGFR mutant type, preoperative and postoperative therapies, medication time, and timing of surgery; 3) ORR, SRR (defined as the percentage of patients who underwent surgery after neoadjuvant therapy), downstage rate, R0 resection rate (defined as the percentage of patients who underwent radical resection after neoadjuvant therapy), pCR rate (defined as the proportion of patients with no tumor cells in all pathologic samples surgically resected after neoadjuvant therapy); 4) DFS (defined as the time from surgery to tumor recurrence or death from any cause), PFS (defined as the time from the neoadjuvant treatment to disease progression or death from any cause), and OS (defined as the time from neoadjuvant treatment to the date of death or the last follow-up); and 5) AEs during neoadjuvant treatment and the perioperative period.

### Statistical Analysis

Statistical analyses were performed with Stata 15.0 software (StataCorp LLC, College Station, TX, USA). The data of the main outcomes of each study were pooled, which included the ORR, SRR, downstage rate, R0 resection rate, pCR rate, median PFS, median OS, and incidence rate of AEs. Statistical heterogeneity among the studies was detected with the *I^2^* statistic. If the probability (*p*) value was ≤ 0.05 or *I^2^* > 50% indicated significant heterogeneity, a random-effects model (DerSimonian-Laird method) was used. Otherwise, a fixed-effects model (inverse-variance method) was used.

### Sensitivity Analysis and Publication Bias

Sensitivity analyses were performed for the ORR results based on the leave-one-out approach. The potential for publication bias in the reported ORR values was assessed using funnel plots, with the appropriate accuracy intervals.

## Results

### Study Population and Patient Characteristics

**Figure 1:**
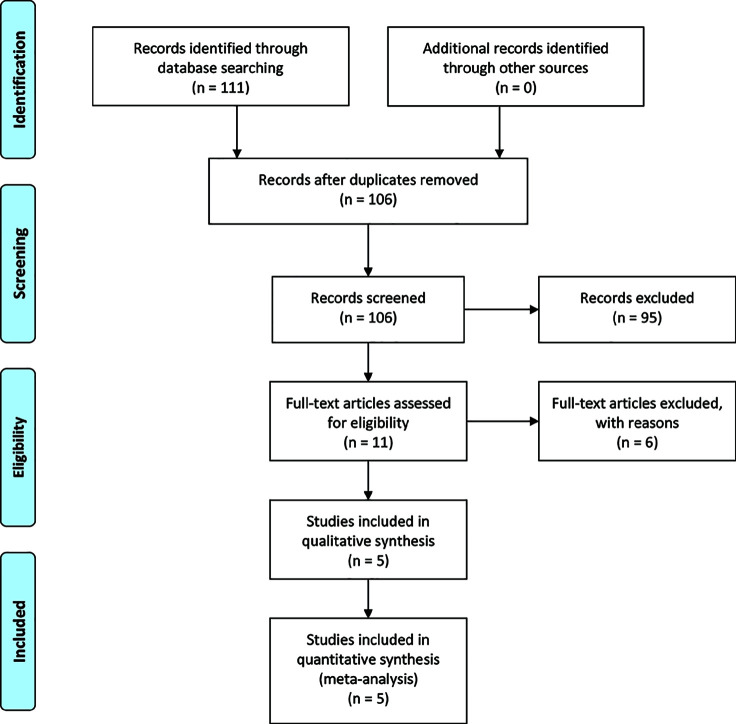
A flow diagram of the literature search and selection process.

A PRISMA flow diagram of the literature search process is shown in **Figure 1**. A total of five, phase II, prospective, clinical trials involving 124 patients with resectable or potentially resectable EGFR-mutant NSCLC were included in this pooled analysis. Among the five studies, three were single arm trials and two were RCTs. Three studies included patients with only stage IIIA disease (5, 15, 16), while the other two included patients with stages IA–IIB or II–IIIA disease without further stratification (**Table 1**) (18, 19). The data of 68 patients with stage IIIA disease from three studies were extracted as a subgroup for independent analysis (5, 16, 17).

**Table 1:** Characteristics of included studies.

Study	Zhong (5)	Xiong (16)	Zhong (17)	Rizvi (18)	Zhang (19)
Enrollment years	2011–2017	2011–2014	2008–2011	2004–2008	2013–2015
Case number	37	19	12	21	35
Clinical stage	IIIA	IIIA	IIIA	IA–IIB	II–IIIA
Preoperative Tx	Erlotinib	Erlotinib	Erlotinib	Gefitinib	Gefitinib
Tx duration (day)	42	56	42	21	42
Postoperative Tx	Erlotinib (1 year)	chemotherapy	NR	Gefitinib (2 years)	chemotherapy
ORR	54.1%	42.0%	58.3%	81.0%	54.5%
Operation time^#^	One week	NR	One week	Two days	NR
Downstage rate	10.8%	21.1%	16.7%	NR	20.0%
Surgery rate	83.8%	73.7%	50.0%	100%	94.3%
R0 rate	73.0%	68.4%	25.0%	NR	82.8%
pCR rate	0	0	NR	NR	12.1%
DFS (mo)	NR	10.3	8.6	NR	33.5
PFS (mo)	21.5	11.2	6.9	NR	NR
OS (mo)	45.8	51.6	14.5	NR	NR
SAEs	0	10.5%	16.7%	NA	0

Tx, treatment; ^#^the time from drug discontinuance to surgery; SA, single arm; RCT, randomized controlled trial; ORR, objective response rate; pCR, pathologic complete response; DFS, disease-free survival; PFS, progression-free survival; OS, overall survival; NR, not report; NA, not available; SAEs: grade3/4 adverse events during preoperative therapy.

The characteristics of patients in the included studies are summarized in **Table 2**. All patients had an ECOG performance status score of 0–1 point, while 68 (54.8%) were treated with neoadjuvant erlotinib and 56 (45.2%) with neoadjuvant gefitinib. The median medication time was 42 (range, 21–56) days. The median time of response evaluation was 45 (range, 42–56) days.

**Table 2:** Characteristics of included patients (n=124).

Characteristics	Case number (%)
ECOG 0–1	124 (100%)
Age median (range)	60 (57–67)
Sex	
Male	35 (28.2%)
Female	68 (54.4%)
Unknown	21 (17.4%)
Smoke status	
Ever	25 (20.2%)
Never	66 (53.2%)
Unknown	33 (26.6%)
Histology	
Adenocarcinoma	62 (51.7%)
Non-adenocarcinoma	6 (5.0%)
Unknown	52 (43.3%)
Clinical stage	
IA–IIB (17, 18)	29 (23.4%)
IIIA	95 (76.6%)
Mutation status	
Exon 19 deletion	68 (54.8%)
Exon 21 L858R	56 (45.2%)
Preoperative Tx	
Erlotinib	68 (54.8%)
Gefitinib	56 (45.2%)

### ORR, SRR, and Postoperative Outcomes

**Figure 2:**
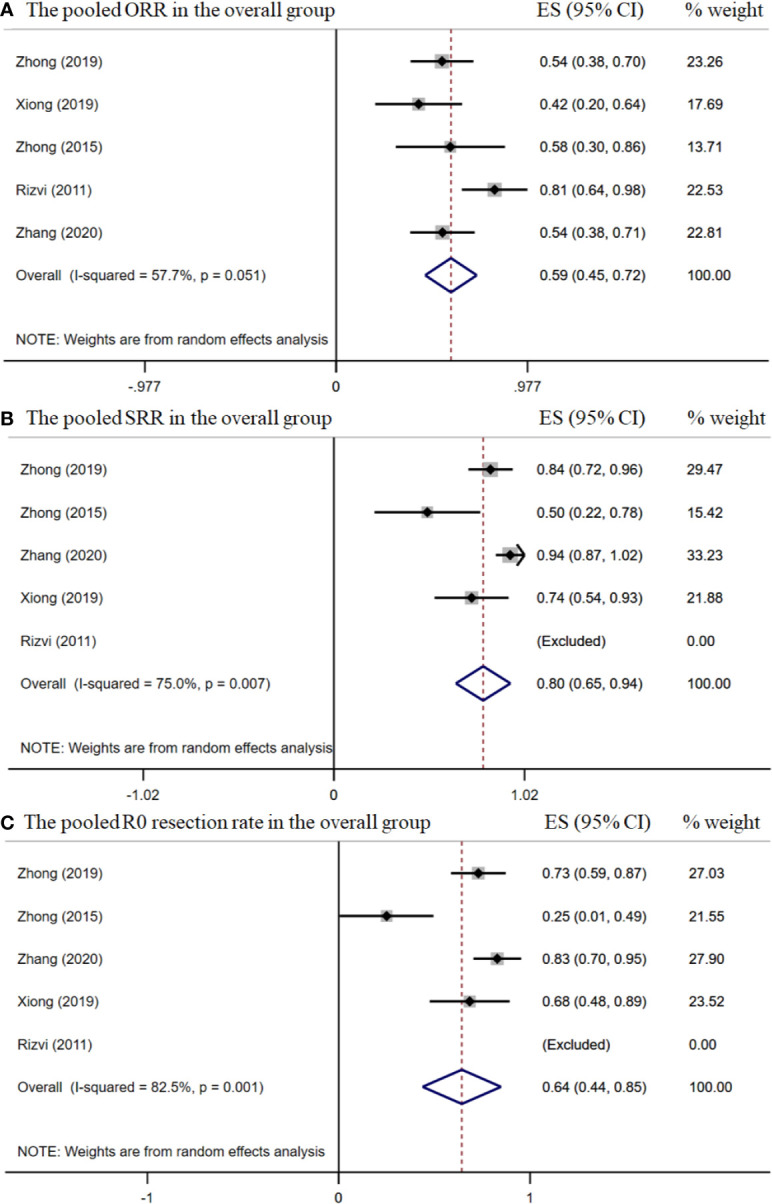
The pooled efficacy rates in the overall group. The ORR **(A)**; SRR **(B)**; and R0 resection rate **(C)**.

**Figure 3:**
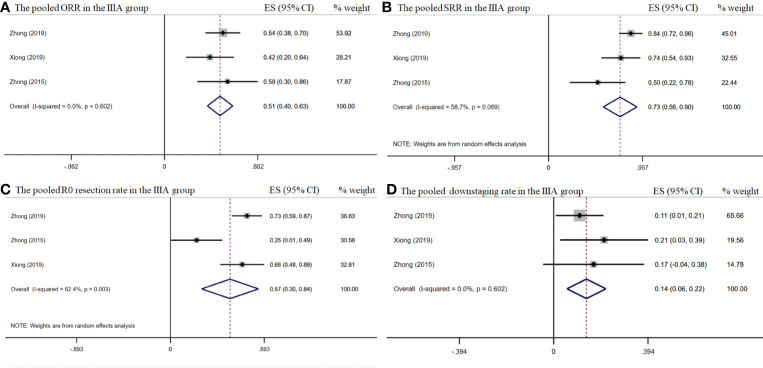
The pooled efficacy rates in the stage IIIA subgroup. The ORR **(A)**; SRR **(B)**; R0 resection rate **(C)**; and downstaging rate **(D)**.

**Figure 4:**
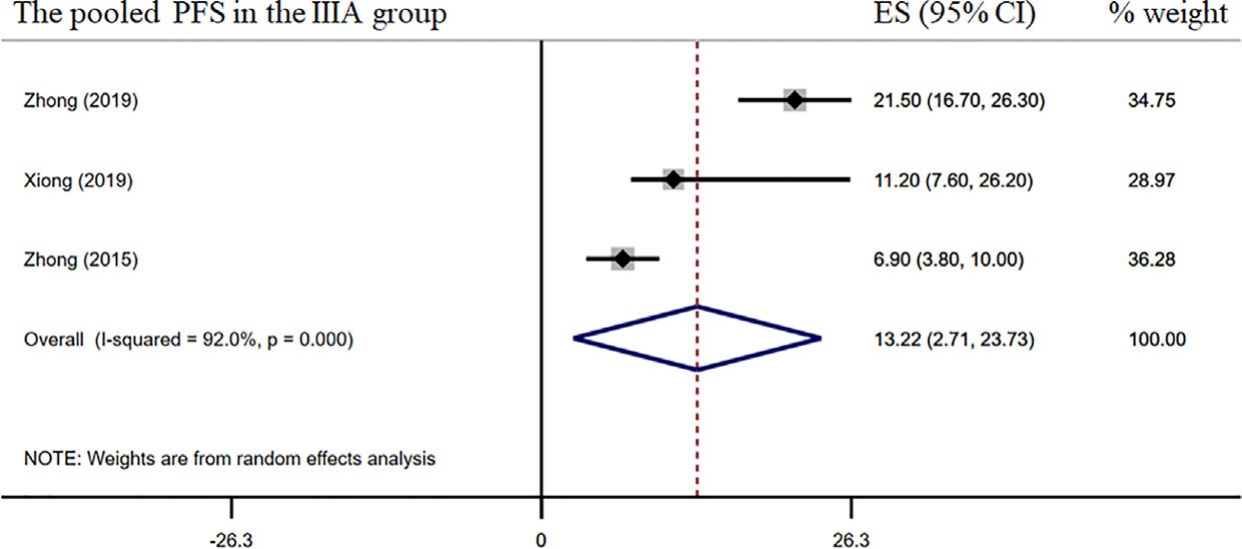
Median PFS of the stage IIIA subgroup.

The pooled overall ORR was 58.5% [95% confidence interval (CI), 45.5%–71.8%] (**Figure 2A**). The surgical resection and R0 rates were 79.9% (95% CI, 65.3%–94.5%) and 64.3% (95% CI, 43.8%–84.8%), respectively (**Figures 2B–C**). In the stage IIIA subgroup, the pooled ORR was 51.4% (95% CI, 39.7%–63.2%) (**Figure 3A**), while the surgical resection and R0 rates were 72.9% (95% CI, 55.7%–90.1%) and 56.8% (95%CI, 29.8%–83.8%), respectively (**Figures 3B–C**). The downstaging rate was 14.0% (95% CI, 5.6%–21.8%) (**Figure 3D**), the pCR rate extracted from two studies was 0.0%, the pooled median PFS was 13.2 months (95% CI, 2.7–23.7) (**Figure 4**), and the pooled median OS was 41.9 months, which was calculated using a weighted average of single study medians because of insufficient data of the 95% CI values (20).

### Safety

The most common AEs observed during neoadjuvant treatment are listed in **Table 3**. The most common AEs were rash and diarrhea. The pooled incidence rates of any grade and grade ≥ 3 rash were 54.9% and 14.7%, respectively. The pooled incidence rate of any grade diarrhea was 14.7%. No grade ≥ 3 diarrhea was reported. The pooled incidence rates of any grade and grade ≥ 3 hepatotoxicity were 7.7% and 5.3%, respectively. Other AEs, including paronychia, stomatitis, and leukopenia, etc., were reported by limited studies (**Table 3**).

**Table 3:** The main toxicity of neoadjuvant EGFR-TKI therapy.

	subcutaneous tissue disorders				Hematologic	Gastrointestinal	Hepatorenal	
	Rash		Paronychia	Stomatitis	Leukopenia	Diarrhea	Abnormal liver function	
	All grade	≥3 grade	All grade	All grade	≥3 grade	All grade	All grade	≥3 grade
Studies^*^	4	4	2	1	1	4	4	4
Patients^*^	103	103	72	37	19	103	103	103
Events	65	2	3	4	1	26	2	1
pooled incidence rates (%)	54.9	14.7	3.8	10.8	5.3	14.7	7.7	5.3
Range (%)	30.3–79.6	2.7–26.8	0–8.2	NA	NA	2.7–26.8	0–16.6	NA

^*^Number of studies reporting this toxicity and number of patients included in these studies. NA, Not Applicable.

The postoperative complications reported by four studies are listed in **Table 4** (5, 16, 17, 19). The postoperative complications reported by two or more studies included lung infection, arrhythmia, and pneumothorax, but there were no actual concrete data. Other postoperative complications included poor incision healing, chest tube drainage for > 7 days, postoperative bleeding, chylothorax, and pulmonary artery injury, but without concrete data. There was no report of increased operative difficulty or perioperative death.

**Table 4:** Postoperative complications.

Study^*^	Lung infection	Sinus tachycardia or arrhythmia	Chylothorax	Poor incision healing	Lung infection or left-sided pneumothorax	Chest tube drainage for >7 days	Postoperative bleeding	Pulmonary artery injury
Zhong (5)	2 (6.5%)	2 (6.5%)		2 (6.5%)	1 (3.2%)	1 (3.2%)		
Zhong (17)							1 (16.7%)	
Zhang (19)			4 (12.1%)					
Rizvi (18)	Y	Y			Y			Y

^*^Studies reported the result of postoperative complications; Y, Study just reported the events without concrete data.

### Sensitivity Analysis and Publication Bias

**Figure 5:**
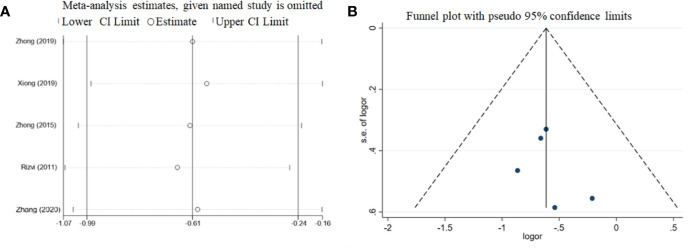
Sensitivity analyses **(A)** and funnel plot **(B)** of the ORR among the included studies.

The results of the leave-one-out sensitivity analyses for the ORR are summarized in **Figure 5A**. The estimated ORR of each study was similar to the pooled ORR value and 95% CI. Potential publication bias was assessed using funnel plots with ORR. The funnel plots were symmetrical, indicating no publication bias (**Figure 5B**).

## Discussion

Neoadjuvant chemotherapy is now an acceptable treatment approach for resectable or potentially resectable NSCLC (21). However, the role of neoadjuvant targeted therapy remains unclear due to the lack of prospective phase III RCTs. Our pooled analysis indicated that neoadjuvant EGFR-TKI therapy may provide a feasible treatment modality for patients with resectable or potentially resectable EGFR-mutant NSCLC, with satisfactory surgical outcomes and low toxicity. Although further phase III clinical trials are needed to confirm these findings, especially whether neoadjuvant EGFR-TKI treatment can improve survival of such patients, several controversial questions were addressed.

The first question is whether neoadjuvant EGFR-TKI was more effective than neoadjuvant chemotherapy for EGFR-mutant NSCLC patients. If the group of patients being treated had advanced unresectable or metastatic NSCLC, this question was not difficult to answer. As for patients with advanced NSCLC with EGFR-sensitive mutations, more than a dozen phase III RCTs studies have reached a consistent conclusion that, as compared to platinum-based doublet chemotherapy, EGFR-TKIs significantly improved the median PFS (9–20 months) and ORR (60%–80%) (8–11). In the phase II EMERGING (CTONG 1103) study, which included a total of 72 patients with stage IIIA-N2 EGFR-mutated NSCLC and compared neoadjuvant erlotinib with neoadjuvant chemotherapy of gemcitabine plus cisplatin, the primary endpoint of ORR was 54.1% (95% CI, 37.2%–70.9%) versus 34.3% (95% CI, 17.7%–50.8%), respectively, with an odds ratio of 2.26 (95% CI, 0.87–5.84; *p* = 0.092) (5). Although the difference was not statistically significant, the ORR tended to be higher for neoadjuvant EGFR-TKI treatment than neoadjuvant chemotherapy. In the present study, the pooled ORRs of overall stage I–IIIA patients and stage IIIA subgroup were 58.5% and 51.4%, respectively, both of which were numerically superior to those in previous studies of neoadjuvant chemotherapy (28%–49%) (22–24). Among these neoadjuvant chemotherapy studies, most patients had stage IB–IIIA NSCLC with predominant squamous cell carcinomas or adenocarcinomas treated with three cycles of platinum-based chemotherapy followed by surgical resection. Despite the insufficient sample size of CTONG 1103, the secondary endpoint (PFS) was signiﬁcantly improved. The median PFS was 21.5 months with erlotinib vs. 11.4 months with chemotherapy (HR, 0.39; 95% CI, 0.23 to 0.67; *p* < 0.001). However, there was no significant difference in OS between the two groups (45.8 vs. 39.2 months; HR, 0.77; 95% CI, 0.41–1.45; *p* = 0.417). The limited number of patients and differences in follow-up treatment may be the main reasons for the lack of differences in OS. In the present study, the pooled median OS for neoadjuvant EGFR-TKIs in the stage IIIA group was 41.9 months, which is comparable to the OS results of previous studies of neoadjuvant chemotherapy (median range, 16–55 months) (22–24). Due to the lack of more RCTs, it was unclear whether neoadjuvant EGFR-TKI therapy could improve OS as compared to neoadjuvant chemotherapy. Ongoing large phase III RCTs (e.g., NCT03203590) will further clarify the difference in OS between neoadjuvant chemotherapy and neoadjuvant EGFR-TKI.

Surprisingly, the higher ORR for neoadjuvant EGFR-TKI treatment did not appear to be associated with a remarkable improvement in surgical outcomes. In the CTONG 1103 study, the surgical resection and R0 rates were 83.8% and 73%, respectively, in the preoperative EGFR-TKI group, and 68.6% and 62.9%, respectively, in the preoperative chemotherapy group, while the downstaging and pCR rates were only 10.8% and 0%, and 2.9% and 0%, respectively in the two groups. In a study by Zhang et al., neoadjuvant therapy with gefitinib for 35 patients with operable stage II-IIIA NSCLC with EGFR-sensitive mutations led to a pCR of 12.1% (4/33), major pathological response rate of 24.2% (8/33), and an ORR of 54% (18/33) (19). In the present review, data of 68 patients with stage IIIA-N2 NSCLC were extracted from three studies for independent analysis. In this subgroup, the surgical resection and R0 rates were 79.7% and 56.8%, respectively. However, the downstaging and pCR rates were merely 14.0% and 0%, respectively. Numerically, a portion of surgical outcomes in the present study, especially the downstaging and pCR rates, was inferior to those in previous studies of neoadjuvant chemotherapy (22–24). In a phase III RCT comparing induction chemoradiation with induction chemotherapy, which included 232 patients with stage IIIA-N2 NSCLC, the surgical resection and R0 rates in the induction chemotherapy group were 82% and 81%, respectively, and the downstaging and pCR rates were 53.0% and 16%, respectively (n = 117) (25). In another large RCT involving 354 patients with stage IB–IIIA NSCLC (excluding N2 disease), the surgical resection and R0 rates in the neoadjuvant chemotherapy group were 89.9% and 93%, respectively (22). Consistently, the EGFR mutation status was not elucidated in these previous studies.

In brief, neoadjuvant EGFR-TKI therapy could significantly shrink tumor volume and improve radiological responses, while increasing the curative resection rate. However, this impressive tumor shrinkage effect has not been translated into changes in disease stage or pCR rate. We believe that the spatial heterogeneity within and between tumors may be the main reason for this unexpected result.

The second question is the timing of EGFR-TKI medication for patients with operable NSCLC with EGFR-sensitive mutations, as it remains unclear whether preoperative or postoperative administration of EGFR-TKI, or both is more beneficial for these patients. Indeed, if the patient population is diagnosed with EGFR-mutant NSCLC after surgery, particularly stage IIIa-pN2, the question has been positively answered with adjuvant EGFR-TKI treatment. Several RCTs reported that adjuvant EGFR-TKI therapy significantly improved DFS (28.7 vs. 18.0 months for the ADJUVANT study; 42.4 vs. 21.0 months for the EVAN study, and not reached vs. 20.4 months for the ADAURA study, respectively) as compared with adjuvant chemotherapy or placebo for patients with postoperative stage II-III NSCLC with EGFR-sensitive mutations (12–14), thus providing strong evidence for adjuvant EGFR-TKI therapy. In the present study, the pooled median PFS and OS for neoadjuvant EGFR-TKIs in the stage IIIA group were 13.2 and 41.9 months, respectively, but median PFS values varied between 6.9 and 21.5 months. In the CTONG 1103 study, the erlotinib-treatment group of patients who were intended to receive neoadjuvant erlotinib therapy for 42 days and adjuvant erlotinib therapy for 1 year obtained a median PFS of 21.5 months (5). In a study by Xiong et al., patients who received neoadjuvant erlotinib therapy for 56 days and three cycles of adjuvant chemotherapy achieved a median PFS of 11.2 months (16). In the CSLC0702 study, the median PFS was only 6.9 months (17). This difference might be attributed to inconsistencies in subsequent adjuvant therapies (postoperative chemotherapy vs. adjuvant EGFR-TKIs vs. postoperative radiotherapy etc.). Clinically, for patients with operable EGFR-mutant NSCLC, adjuvant chemotherapy, EGFR-TKI, or a combination of both are currently acceptable treatment options, although the most efficacious remains controversial. For potentially resectable NSCLC, neoadjuvant EGFR-TKI should be considered given the better ORR, SRR, and safety as compared to chemotherapy.

The third question concerns the duration of neoadjuvant EGFR-TKI therapy. In our pooled analysis, the median medication duration was 42 (range, 21–56) days and the efficacy evaluation time was 45 (range, 42–56) days. Of note, for advanced NSCLC, the ORR for neoadjuvant EGFR-TKI was slightly lower than that for first-line EGFR-TKI (58% vs. 62%–70%, respectively). For advanced disease, the efficacy evaluation time commonly ranged between 42 and 56 days (8–11). Different durations of drug exposure might influence efficacy. In the five included studies, the ORR varied from 42% to 81%. Paradoxically, Rizvi et al. reported a medication time of 21 days and ORR of 81% (18), while Xiong et al. reported a medication time of 56 days and ORR of only 42% (16). Obviously, patient characteristics and the neoadjuvant drugs of EGFR-TKIs differed among these studies. The study by Xiong et al. was limited to patients with stage IIIA NSCLC treated with erlotinib therapy, while the study by Rizvi et al. was limited to patients with IA–IIB early-stage NSCLC treated with gefitinib. According to the ORR results and postoperative outcomes, 42 days is a rational medication time for clinical treatment because the effect would not be evaluated prematurely, the delay in surgical intervention would not too long, and toxicities would not obviously increase.

The last question addresses the safety of neoadjuvant EGFR-TKI. Neoadjuvant TKI therapy appears to be generally well tolerated. Similar to the AEs as the first-line treatment for patients with advanced disease, the common side effects were skin rash, diarrhea, and other skin and subcutaneous tissue disorders, as well as hepatotoxicity. The incidence of grade 3/4 adverse events was 5.3% for hepatotoxicity and 14.7% for skin rash. Surgery was no delayed for any patient due to treatment-related AEs (TRAEs). In contrast, TRAEs, including perioperative death and treatment-induced surgery delay, limit the application of preoperative chemotherapy (22, 26). In total, 48%–60% of AEs were grade 3/4 and 6% of TRAEs led to permanent discontinuation of chemotherapy (25, 26).

We were more concerned with surgical difficulties and risks, and intra- and postoperative complications. In the CTONG 1103 study, the types of resection in the erlotinib and chemotherapy groups were lobectomy (64.9% vs. 54.3%), bi-lobectomy (13.5% vs. 14.3%), and pneumonectomy (5.4% vs. 0.0%, respectively). Zhang et al. reported lobectomy in 93.9% of patients and bi-lobectomy in 6.1% (19). The most common postoperative complications were lung infection, arrhythmia, and pneumothorax. No perioperative death, increase in surgical difficulty, or postoperative complications caused by neoadjuvant EGFR-TKI was observed.

There were some limitations to this pooled analysis. Although there was no publication bias, the included studies were all phase II clinical trials with small sample sizes. Furthermore, differences in patient characteristics may have influenced the results. In addition, different medications for different EGFR-TKI types were not stratified, so it remains to be determined whether efficacy differed among the different EGFR-TKIs.

## Conclusion and Future Perspectives

Although data from prospective phase III RCTs evaluating the role of neoadjuvant targeted therapy for patients with EGFR-mutant NSCLC are lacking, the results of this pooled analysis indicated that short-term (median, 42 days; range, 21–56 days) neoadjuvant EGFR-TKI therapy provided a feasible treatment modality for patients with resectable or potentially resectable EGFR-mutant NSCLC, with satisfactory surgical resection and R0 rates (80% and 64.3%, respectively), but modest downstaging and pathological complete response rates (14% and 0%, respectively). The incidence of grade 3/4 toxicity was low. Because the studies included in this pooled analysis were all phase II clinical trials with small sample sizes, further studies with well-designed phase III clinical trials are warranted to confirm the efficacy and safety of neoadjuvant EGFR-TKIs. An ongoing clinical trial (NCT03203590) is investigating the efficacy and safety of gefitinib neoadjuvant targeted therapy and vinorelbine/carboplatin neoadjuvant chemotherapy for resectable stage II-IIIA NSCLC patients with EGFR mutation.

There is an urgent need to explore a more effective neoadjuvant targeted therapy regimen given the modest downgrade and pCR rates for EGFR-TKI alone. Two RCTs showed that first-line treatment with gefitinib plus chemotherapy achieved a significantly higher ORR (84% vs. 67% for the NEJ009 study; 75% vs. 63% for the study by Noronha et al.), longer PFS (median, 20.9 vs. 11.9 months for the NEJ009 study; 16 vs. 8 months for the study by Noronha et al.) and longer OS than gefitinib alone for patients with EGFR-mutant advanced NSCLC (27, 28). Given the strong ORRs and PFS, it is very worthwhile to design clinical trials to validate the feasibility of chemotherapy combined with EGFR-TKI as a neoadjuvant therapy for EGFR-mutant NSCLC.

Because the results of adjuvant osimertinib in the phase III ADAURA study were impressive, a single arm phase II trial (NCT03433469) is ongoing to evaluate the efficacy of osimertinib as a neoadjuvant therapy for patients with surgically resectable (stage I-IIIA) EGFR-mutant NSCLC, and a phase III trial neoADAURA (NCT04351555) is planned to compare neoadjuvant osimertinib, with or without chemotherapy, and chemotherapy alone for resectable NSCLC (29). These prospective clinical studies will confirm whether and what type of EGFR-TKI neoadjuvant treatment can improve survival of patients with EGFR mutations.

## Data Availability Statement

Publicly available datasets were analyzed in this study. The original contributions presented in the study are included in the article/supplementary material. Further inquiries can be directed to the corresponding author.

## Author Contributions

J-TM: conceptualization, methodology, manuscript review, and revision. LS: writing of the original draft. Y-JG: data extraction and collection. JS: data extraction and collection. WJ: software. Y-RW: manuscript review and revision. S-LZ: Software. L-TH: formal analysis. J-ZZ: table editing. C-BH: conceptualization, methodology, and supervision. All authors contributed to the article and approved the submitted version.

## Funding

This study was supported by grants from the 345 Talent Project of Shengjing Hospital.

## Conflict of Interest

The authors declare that the research was conducted in the absence of any commercial or financial relationships that could be construed as a potential conflict of interest.

## Abbreviation

AEs, adverse events; CI, confidence interval; CTONG, Chinese Thoracic Oncology Group; DFS, disease-free survival; EGFR, epidermal growth factor receptor; ESMO, European Society for Medical Oncology; NSCLC, non-small cell lung cancer; ORR, objective response rate; OS, overall survival; PFS, progression-free survival; pCR, pathologic complete response; RCTs, randomized controlled trials; SRR, surgical resection rate; TKI, tyrosine kinase inhibitor; TRAEs, treatment-related adverse events.
